# Adaptogenic Effects of Mushroom Blend Supplementation on Stress, Fatigue, and Sleep: A Randomised, Double‐Blind, and Placebo‐Controlled Trial

**DOI:** 10.1002/brb3.71193

**Published:** 2026-01-15

**Authors:** Ahmad Safiyyu'd‐din Hisamuddin, Faiqah Ramli, Teik Kee Leo, Mohamad Shazeli Che Zain, Mei Szin Wong, Mohd Raili Suhaili, Le Jie Lee, Tze Yan Lee

**Affiliations:** ^1^ Research and Development Department Nexus Wise Sdn Bhd Petaling Jaya Malaysia; ^2^ Bioresource Technology Division, School of Industrial Technology Universiti Sains Malaysia Penang Malaysia; ^3^ Faculty of Medicine, Nursing and Health Sciences SEGi University Petaling Jaya Selangor Malaysia; ^4^ Prima Nexus Sdn. Bhd. Bandar Puteri Puchong Selangor Malaysia; ^5^ Clinical Laboratory Science Section, Institute of Medical Science Technology Universiti Kuala Lumpur Kajang Selangor Malaysia; ^6^ Department of Medical Education Sif Jeffrey Cheah Sunway Medical School, Faculty of Medical and Life Sciences, Sunway University Malaysia

**Keywords:** adaptogen, β‐glucan, mushrooms, stress

## Abstract

**Background/Objectives:**

Medicinal mushrooms have been gaining increasing attention as functional foods; however, scientific evidence from human studies remains limited.

**Methods:**

In this study, 50 participants were randomly assigned to receive either Restake or a placebo. Psychological and physiological parameters were assessed at baseline, 6 weeks, and 12 weeks using validated tools, including the Pittsburgh Sleep Quality Index (PSQI), Visual Analog Scale for Fatigue (VAS‐F), Multidimensional Fatigue Inventory (MFI), State‐Trait Anxiety Inventory (STAI‐S), Perceived Stress Scale (PSS), Hamilton Anxiety Scale (HAM‐A), and Beck Depression Inventory (BDI). Serum biomarkers—cortisol, norepinephrine (NE), melatonin, adrenocorticotropic hormone (ACTH), and C‐reactive protein (CRP)—were analyzed via ELISA.

**Results:**

Anxiety, assessed by STAI‐S and HAM‐A, showed greater reductions in the Restake group at both 6 weeks (STAI‐S: *p* = 0.025; HAM‐A: *p* = 0.002) and 12 weeks (STAI‐S: *p* = 0.011; HAM‐A: *p* = 0.002). Depression (BDI) scores significantly decreased at 6 weeks (*p* < 0.001) and 12 weeks (*p* = 0.008). Fatigue levels showed significant reductions in general fatigue (*p* = 0.043), physical fatigue (*p* = 0.027), and mental fatigue (*p* = 0.043). Restake supplementation led to reductions in sleep quality scores (PSQI) at 6 weeks (*p* = 0.005) and 12 weeks (*p* < 0.001). Biomarker analysis revealed significant reductions (*p* < 0.001) in cortisol and ACTH levels and a decrease in CRP levels (*p* = 0.042). NE levels significantly (*p* = 0.033). Compared to the placebo group, Restake supplementation exhibited an increased morning melatonin trend after 12 weeks of intervention.

**Conclusions:**

Restake supplementation was well tolerated and effectively reduced psychological stress, fatigue, and improved sleep quality without adverse effects.

## Graphical abstract

In a randomized, double‐blind, placebo‐controlled trial, 50 moderately to severely stressed adults received Restake or placebo for 12 weeks. Psychological outcomes, including anxiety, depression, fatigue, perceived stress, and sleep quality, were evaluated alongside stress‐related biomarkers. Restake supplementation significantly reduced anxiety, depression, and multiple fatigue domains, while improving sleep quality over time. Biomarker analysis showed reductions in cortisol, adrenocorticotropic hormone, norepinephrine, and C‐reactive protein, with an increasing trend in morning melatonin levels. The intervention was well tolerated, supporting the potential role of medicinal mushroom supplementation in managing stress‐related symptoms and inflammation.

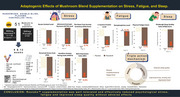

## 1 Introduction

According to the World Health Organization, stress, fatigue, and sleep disturbances are increasingly prevalent in modern societies, affecting a significant proportion of the population. The global prevalence rates have been reported as 28.0% for depression, 26.9% for anxiety, 24.1% for post‐traumatic stress symptoms, 36.5% for stress, 50.0% for psychological distress, and 27.6% for sleep disturbances (Nochaiwong et al. 2021). Chronic stress leads to disruptions in homeostasis, often manifesting as persistent fatigue and poor sleep quality. These conditions often coexist and collectively impair physical and cognitive performance, leading to a diminished quality of life and increased healthcare burdens (Panossian and Wikman 2009, Kelly et al. 2017).

Adaptogens are natural substances that help the body adapt to physical, chemical, and biological stressors by enhancing resilience and restoring balance. They primarily act by modulating the hypothalamic‐pituitary‐adrenal (HPA) axis and regulating key stress mediators, such as cortisol and ACTH (Panossian et al. 2009; Salve et al. 2019). Additionally, adaptogens support homeostasis by influencing neurotransmitter pathways, including serotonin and dopamine, while exerting anti‐inflammatory effects through cytokine regulation (Wagner et al. 2013). These multifaceted mechanisms contribute to their ability to protect against stress‐induced damage and improve overall well‐being.

Individuals experiencing depression, fatigue, and lack of sleep often struggle to cope, resulting in a diminished quality of life, and many still lack access to effective care (Hohls et al. 2021; Scott et al. 2021; World Health Organization 2025; Wang et al. 2007). Prescription medications can be effective but are frequently limited by side effects and acceptability, and they do not work for everyone (Cipriani et al. 2018). Traditional and modern medicine, have therefore explored complementary options for these interconnected issues (Picheta et al. 2024; Dang et al. 2024). This underscores the need for safer, natural alternatives that can holistically improve stress resilience, energy levels, and sleep quality, including phytotherapy for mood disorders and depression (Picheta et al. 2024). Among natural remedies, medicinal mushrooms are discussed as potential adaptogenic agents that support stress resistance and homeostasis (Panossian and Wikman 2020). Their putative adaptogenic effects are attributed in part to polysaccharides such as β‑glucans, which modulate immune function and influence oxidative stress and inflammatory pathways (Ventura‑Sobrevilla et al. 2021; Daou and Zhang 2023; Wu et al. 2021; Cör Andrejč et al. 2022).

Restake is a proprietary blend of five medicinal mushrooms: *Hericium erinaceus* (Lion's Mane), *Cordyceps militaris*, *Ganoderma lucidum* (Reishi), *Lentinula edodes* (Shiitake), and *Grifola frondosa* (Maitake). *H. erinaceus*, *G. frondosa*, and *C. militaris* have been reported to alleviate stress or depressive‑like behaviors in clinical and in vivo studies, in part by modulating BDNF expression, serotonergic pathways, and AMPA receptor signaling, which are central to neurogenesis, mood regulation, and synaptic plasticity (Hoh et al. 2019; Chiu et al. 2018; Li et al. 2015; Zhang et al. 2016; Chen et al. 2021). Similarly, *G. lucidum*, *L. edodes*, and *H. erinaceus* demonstrate anti‑fatigue activity by enhancing glycogen storage, optimizing energy metabolism, and exerting antioxidant and anti‑inflammatory effects that together support physical endurance and recovery (Chen et al. 2024; Wang et al. 2014; Hou et al. 2019; Hirsch et al. 2017; Kim et al. 2020; Sun et al. 2009). *G. lucidum* and *H. erinaceus* have also been shown to improve sleep‑related outcomes via gut‑microbiota modulation and serotonin‑involved pathways in animal and human studies, aligning with observed links between gut composition and sleep behavior (Zhong et al. 2021; Qin et al. 2024; Hoh et al. 2019). In some models, dopamine systems are modulated alongside improved sleep phenotypes, suggesting potential roles in sleep regulation, though directionality and REM‑specific effects vary by study (Harada et al. 2024). Randomized and controlled human studies suggest adaptogenic mushrooms can modestly improve stress, fatigue, sleep, and wellbeing, including *H. erinaceus*, with small benefits on mood and stress in young adults (Docherty et al. 2023). *G. lucidum* plus Ophiocordyceps improving training‐related recovery markers in athletes (Rossi et al. 2014). *G. lucidum* spore powder reduces cancer‐related fatigue in patients on endocrine therapy (Zhao et al. 2012). Meta‐analytic evidence that fungal β‐glucans reduce subjective fatigue and improve vigor and mood (Muroya et al. 2025) and recipe‐dependent anti‐fatigue and sleep‐aiding effects of *G. lucidum* extracts (Li et al. 2024)

A key active compound in Restake is β‐1,3.1,6‐Glucans (β‐glucan), a polysaccharide known for its powerful immunomodulatory and anti‐inflammatory properties. A study conducted by Richter et al. (2015) found that β‐glucan supplementation in cancer patients reduces CRP and cortisol levels, helping to alleviate inflammation and stress‐related fatigue through immune modulation. Individual experience of fatigue usually has an imbalance autonomous nervous system and elevated inflammatory markers, which can be influenced by increased norepinephrine levels and CRP levels (Son et al. 2016). Therefore, the present study would like to assess the efficacy of Restake in ameliorating fatigue and stress via autonomous balance and anti‐inflammatory activities. Additionally, β‐glucan alleviates stress by reducing ACTH and cortisol levels, enhancing immune response, and stimulating BDNF synthesis in the hippocampus through the ERK1/2 pathway. In an open label clinical study, consumption of β‐glucan significantly showed improvements in sleep pattern and quality in autistic children (Raghavan et al. 2022). That said, the synergistic effects from these medicinal mushroom extracts, together with the potent anti‐inflammatory activity of β‐glucan, may function as adaptogens by modulating the hypothalamic–pituitary–adrenal (HPA) axis and reducing systemic inflammation. This dual action is expected to restore stress homeostasis, thereby improving mood, resilience, and overall psychological well‐being.

The primary objective of this study is to evaluate the effect of Restake supplementation on stress reduction, assessed primarily through changes in serum cortisol levels. Secondary objectives include evaluating the effects of Restake on anxiety, depression, fatigue, and sleep quality, as well as its overall adaptogenic and emotional well‐being effects. Additional biomarkers, including ACTH, norepinephrine (NE), melatonin, and CRP, are examined to further characterize the adaptogenic response. Validated questionnaires—including PSQI, VAS‐F, MFI, STAI‐S, PSS, HAM‐A, and BDI—are used to assess stress, fatigue, sleep quality, and emotional well‐being, providing a comprehensive evaluation of the functional and safety effects of Restake. These questionnaires were chosen for their reliability in assessing stress, fatigue, sleep quality, and emotional well‐being, providing a comprehensive evaluation of the adaptogenic and safety effects of Restake.

## 2 Materials and Methods

### 2.1 Preparation of Mushroom Blend and Placebo and Determination of β‐Glucan Level

The mushroom blend at an optimized ratio (Supplementary Data 1, Table 1) was extracted using ultrasonic assisted extraction to maximize the adaptogenic effects of Restake. The extract was subjected to Nutriseal microencapsulation technology, which enhances the solubility and stability of Restake, ensuring its efficacy. This holistic approach in formulation ensures a high standardization of β‐glucan content of more than 30% (β‐Glucan Assay Kit, K‐YBGL, Megazyme) and more than 30% of total polysaccharides (phenol‐sulfuric acid assay). The placebo capsules contained the same excipients as Restake (beta cyclodextrin and arabic gum) but without the mushroom extract. All analytical methods were validated for selectivity, accuracy, and precision. A continuous quality control program was initiated to monitor and maintain the quality of the product.

**TABLE 1:** Baseline demographic details of participants.

Measurements	Placebo (n = 25)	Restake (n = 25)	p‐value
Age	41 ± 6.5	42 ± 6.0	0.596^a^
Gender			
Male (n)	12	8	0.386^b^
Female (n)	13	17	
BMI (kg/m2)	28.8 ± 7.5	28.3 ± 5.9	0.791^a^
BP systolic (mmHg)	120.1 ± 11.7	119.6 ± 17.9	0.913^a^
BP diastolic (mmHg)	79.2 ± 8.5	77.7 ± 11.8	0.696^a^
Pulse	80.7 ± 14.0	79.6 ± 9.4	0.952^a^

*Note*: ^a^T‐test

^b^ Chi‐square test.

### 2.2 Study Design

The study was a double‐blind, randomized, placebo‐controlled trial. This study protocol was approved by the SEGi Research Ethics Committee (SEGiEC/StR/FOM/379/2024‐2025) and was conducted in accordance with the Declaration of Helsinki and Good Clinical Practice Guidelines. Participants were recruited between June and November 2024 across Klang Valley, Selangor, Malaysia.

All participants provided written informed consent to join the study before inclusion. The information about the study was presented to the study participants in Malay and English languages by local regulations. The patient information sheet described the study procedures, the aims, expected benefits, and potential risks. Patients were evaluated by a physical examination performed by an investigator, and appropriate lab tests were conducted. Eligible participants were aged between 22 and 55 years and were reported to have at least three stress‐related symptoms, such as headaches, palpitations at rest, frequent sleep disturbances, or feelings of exhaustion. The age range was selected to ensure optimal homogeneity and include adults most likely to present stress and fatigue‐related symptoms without the confounding influences of adolescent developmental stress (Tsigos and Chrousos 2002; Noushad et al. 2021). Eligibility was further assessed with a score of moderate to severe (40 to 60) on the STAI‐S and a minimum score of 14 on the PSS. Participants also had to demonstrate moderate to severe chronic fatigue symptoms with MFI scores of 7 or higher in the subscales for general, physical, or mental fatigue.

Exclusion criteria were established to minimize confounding factors and ensure participant safety. Individuals with a history of significant medical conditions, including cardiac, hepatic, renal, neurological, or gastrointestinal disorders, were excluded. Pregnant or breastfeeding women were not eligible for participation. The study also excluded those currently taking medications or natural supplements that could interfere with sleep or stress biomarkers or who were diagnosed with sleep disorders, such as obstructive sleep apnea or restless leg syndrome, from the intervention. Lifestyle factors were also considered, with high caffeine intake (more than three cups of coffee or equivalent daily) and excessive alcohol consumption (more than 14 standard drinks per week) serving as exclusion criteria. Patients consented to the study and underwent randomization when inclusion criteria were met. A power analysis was conducted to determine the sample size required to detect a clinically significant difference in the primary outcomes between the treatment and placebo groups. Assuming a medium effect size (Cohen's d = 0.5) from clinical studies on adaptogens and medicinal mushrooms (Salve et al. 2019; Vigna et al. 2019), a significance level (alpha) of 0.05, and a power of 0.80, a sample size of 40 participants (20 per group) was determined to be sufficient. After accounting for a 20% dropout rate, 51 participants were recruited, provided monetary compensation, and insured for study participation.

### 2.3 Intervention

The placebo and Restake capsules were identical in appearance, provided and manufactured by Nexus Wise Sdn Bhd in a GMP‐certified facility. Restake (500 mg) contains 25% of proprietary mushroom blend extract and is standardized with >30% β‐glucan and >30% total polysaccharides (Supplementary Data 2, Table 1). The placebo capsule contained the same excipients as the active capsules (beta cyclodextrin and arabic gum). Labels were identical with a code and were maintained by the sponsor to keep investigators blind and to facilitate code breaking in the case of adverse events. All participants were instructed to take 2 capsules with food for 12 weeks upon visits. Overall medication compliance was monitored and measured by reported pill‐count at weeks 6 and 12. Participants attended three visits: baseline, mid‐intervention (6 weeks later), and post‐intervention (12 weeks).

### 2.4 Efficacy and Safety Outcomes

A trained phlebotomist collected fasted venous blood samples from each participant during the baseline, mid‐intervention, and post‐intervention visits. Serum biomarkers (cortisol, NE, CRP, ACTH, and melatonin) were quantified using ELISA kits (Elabscience Biotechnology Co., Ltd. [Wuhan, China]). The biomarker levels were measured at baseline, mid‐intervention, and post‐intervention visits, respectively. Blood samples were collected in the morning of each visit, centrifuged, and stored at −80°C until analysis. Clinical safety and tolerability of the interventions were measured by analyzing the significant changes in the vital parameters and the biochemical parameters assayed, including liver function, renal function, hematological indices, electrolyte levels, and blood glucose level. Assessment of the adverse event reports was also considered as part of the safety evaluation.

The participants were interviewed face‐to‐face by well‐trained enumerators to collect the required information, such as sociodemographic factors and self‐reported medical history, using standardized questionnaires such as PSQI, VAS‐F, MFI, STAI‐S, PSS, HAM‐A, and BDI. The data collected from the questionnaires were then scored according to the established guidelines for each test, and the results were used to evaluate the changes over time in sleep quality, fatigue, stress, and anxiety levels across the different time points (baseline, mid‐intervention, and post‐intervention).

### 2.5 Outcome Measures

#### 2.5.1 Primary Outcome

Stress and anxiety were measured using specific biomarkers, which are serum cortisol and ACTH, as well as STAI‐S, PSS, HAM‐A, and BDI. The STAI‐S questionnaire measures short‐term changes of state anxiety, reflecting transient stress responses (Spielberger et al. 1983). HAM‐A is a clinician‐rated scale that assesses anxiety severity, including somatic and psychic symptoms covering physical and emotional anxiety components (Hamilton 1959). BDI is a self‐report scale that measures depressive symptoms, including emotional, cognitive, and physical manifestations of depression. It was included to evaluate the mood‐regulating effects of Restake (Beck et al. 1961). The PSS scale assesses perceived chronic stress by measuring how unpredictable, uncontrollable, and overwhelming respondents find their lives (Cohen et al. 1983).

#### 2.5.2 Secondary Outcome

Specific biomarkers used to evaluate fatigue and sleep include NE, CRP, and melatonin level. The VAS‐F scale measures subjective fatigue intensity, capturing momentary fatigue levels (Lee et al. 1991), while the MFI assesses general, physical, and mental fatigue, motivation, and activity (Smets et al. 1995). Additionally, PSQI provides a comprehensive assessment of sleep quality, latency, duration, efficiency, disturbance, use of sleep medication, and daytime dysfunction, which are often affected by stress and fatigue (Buysse et al. 1989).

### 2.6 Statistical Analysis

Statistical Package for Social Science (SPSS) version 26 software was used to conduct all statistical analyses at a significance level of *p* < 0.05. Baseline data for demography, questionnaire scores, and biomarkers were compared between the two groups of Placebo and Restake. Data normality was tested using the Shapiro‐Wilk test. One‐way repeated measures ANOVA was used to compare the within group effect (Placebo and Restake) at different timepoints (baseline, Week 6, and Week 12) of interventions, with the post hoc test of Bonferroni. Non‐parametric tests were used for ranking data and non‐normal data, which is the Mann‐Whitney U test. Between groups analysis of percentage changes from baseline to Week 12 was conducted using the independent sample t‐test. All analyses were performed on the per‐protocol population, with participants included if they completed at least 80 percent of study capsules.

Changes in each questionnaire's scores and biomarker levels between the baseline and Week 12 after treatment with Restake or placebo will be calculated to assess the primary outcome according to the following equations:

$$C_{\mathrm{RTK}}=X_{\mathrm{RTK}\left(W12\right)}-X_{\mathrm{RTK}\left(\mathrm{BL}\right)}/X_{\mathrm{RTK}\left(W12\right)}$$

$$C_{\mathrm{PL}}=X_{\mathrm{PL}\left(W12\right)}-X_{\mathrm{PL}\left(\mathrm{BL}\right)}/X_{\mathrm{PL}\left(W12\right)}$$

where C is the changes in score/biomarkers level, X is the score/biomarkers level, W12 is the mean score at Week 12, BL is the baseline, RTK is Restake and PL is placebo.

## 3 Results

### 3.1 Demographic Data and Baseline Characteristics

A total of 50 participants were enrolled in the study, with 40% males and 60% females. The mean age of participants was 41 years for the Placebo and 42 years for the Restake group. Baseline assessments confirmed that participants exhibited moderate to severe stress and fatigue levels, as indicated by STAI‐S, PSS, HAM‐A, and BDI scores, consistent with the predefined inclusion criteria. Fatigue severity, as assessed by VAS‐F and MFI (General, Physical, and Mental Fatigue), further confirmed that participants met the study eligibility requirements. Sleep quality, assessed by PSQI, indicated poor sleep patterns at baseline. Baseline biomarker levels, including cortisol, ACTH, NE, melatonin, and CRP, were recorded for comparison throughout the study. There were no statistically significant differences in any baseline demographic or clinical characteristics between the placebo and Restake groups, ensuring a well‐matched participant population.

**FIGURE 1:**
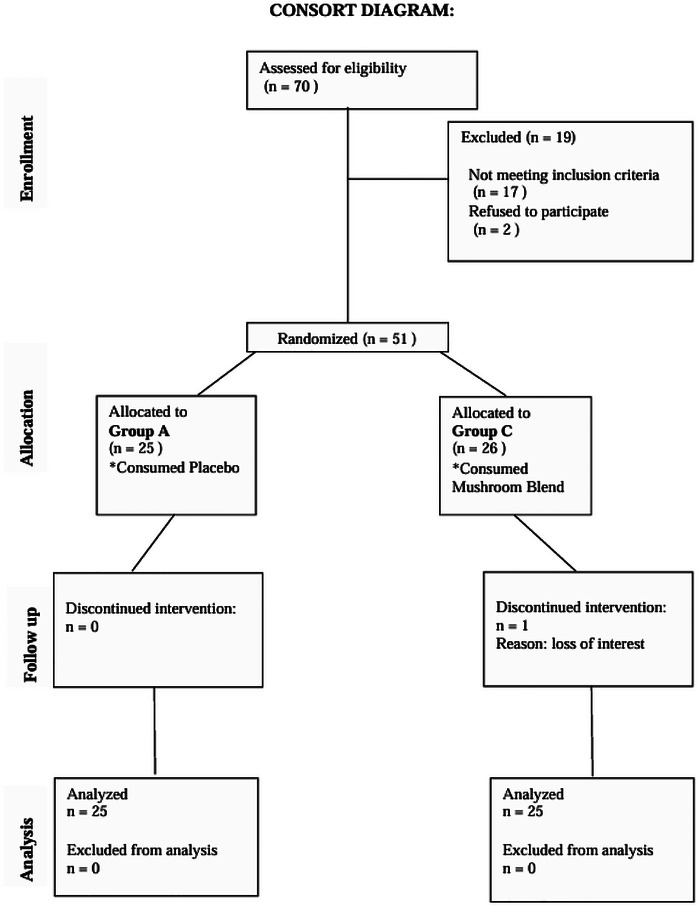
Study CONSORT Flow Diagram. Consort diagram of patients eligible, recruited, numbers followed up and included in analysis.

A total of 70 participants were screened for eligibility, of whom 19 were excluded; 17 did not meet the inclusion criteria, and 2 declined to participate. The remaining 51 participants were randomized into two groups, with 25 participants assigned to the placebo group and 26 participants assigned to the Restake group. During the intervention period, one participant from the Restake group discontinued due to loss of interest. At the end of the study, 25 participants in each group successfully completed the trial and were included in the final analysis. At the end of the study, the majority of participants demonstrated high adherence, with 90% consuming more than 80% of the capsules. Participants with compliance below 80 percent were excluded from the final analysis. No significant differences in compliance rates were observed between the Placebo and Restake groups, indicating consistent adherence across both intervention arms Figure 1.

### 3.2 Primary Outcome

**FIGURE 2:**
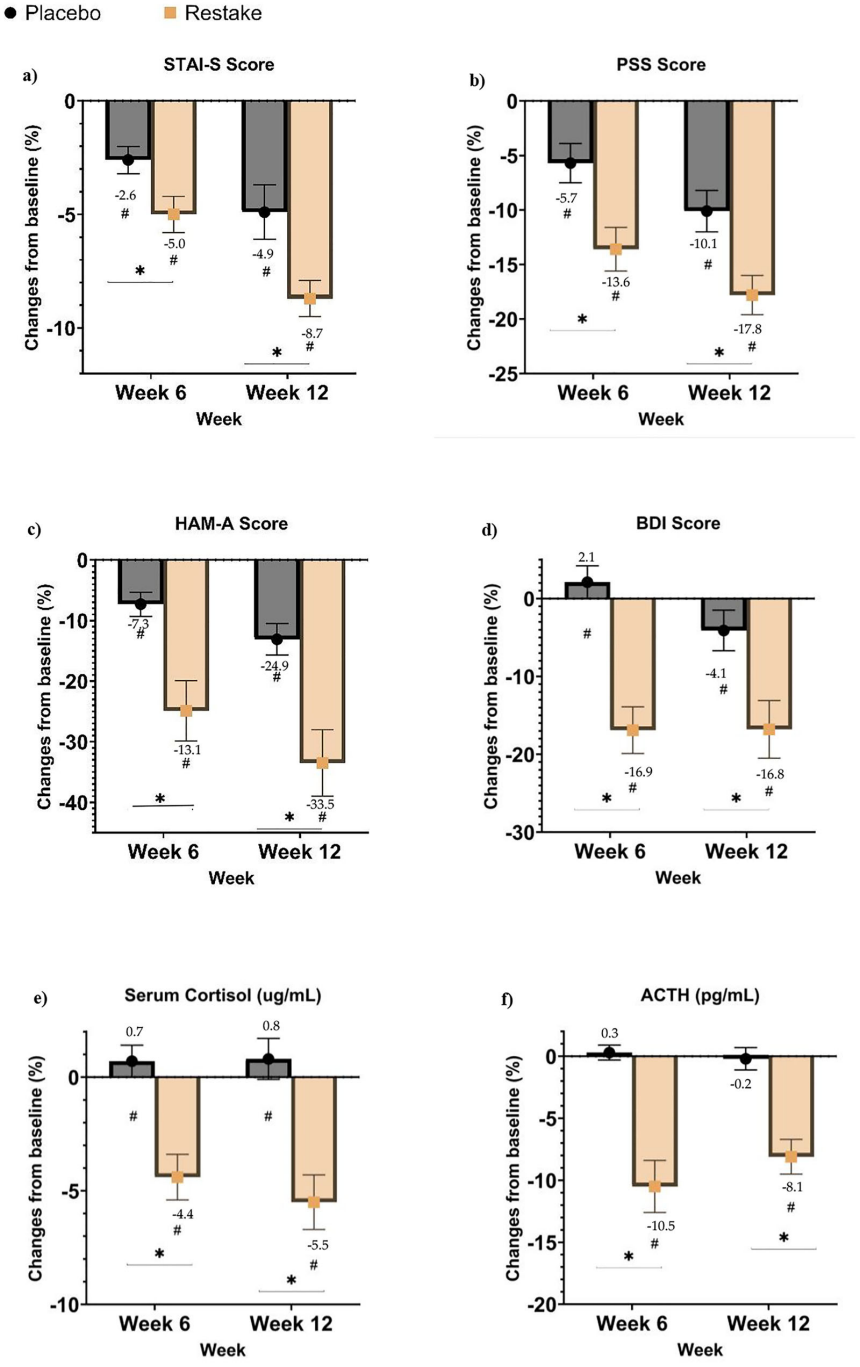
Percentage changes from baseline in (a) STAI‐S, (b) PSS, (c) HAM‐A, (d) BDI, (e) serum cortisol, and (f) ACTH level. * Indicates a significant difference between the Placebo and the Restake groups, while # indicates a significant difference within the group.

The primary outcome of this study is to evaluate the effect of Restake on stress reduction, measured through specific biomarkers (serum cortisol and ACTH) and self‐reported questionnaires, STAI‐S, HAM‐A, PSS, and BDI. Repeated measure ANOVA shows a statistically significant reduction in the STAI‐S, PSS, HAMA, BDI, cortisol level, and ACTH over time in both Restake and placebo (*p* < 0.05) as tabulated in Table 2. Independent sample T‐test analysis revealed that the Restake group showed greater reductions in state anxiety (STAI‐S) compared to the placebo group. These are depicted by asterisks in Figure 2. At 6 weeks, anxiety levels decreased by 5.0% with Restake, while the placebo group showed a smaller reduction of 2.6% (*p* = 0.025). By 12 weeks, the reduction with Restake reached 8.7%, whereas the placebo group experienced a 4.9% decrease (*p* = 0.011). Anxiety measured by the HAM‐A showed greater reductions in the Restake group, with decreases of −24.9% at 6 weeks and ‐33.5% at 12 weeks, compared to reductions of −7.3% and −13.1%, respectively, in the placebo group (6 weeks, *p* = 0.002; 12 weeks, *p* = 0.002). Depression, as assessed by the BDI, increased in the placebo group by +2.1% at 6 weeks and +4.1% at 12 weeks, while the Restake group showed significant reductions of −16.9% and −16.8%, respectively (6 weeks, *p* < 0.001; 12 weeks, *p* = 0.008). PSS demonstrated significant reductions in the Restake group, with decreases of −13.6% at 6 weeks and ‐17.8% at 12 weeks, compared to smaller reductions of −5.7% and −10.1%, respectively, in the placebo group (6 weeks, *p* = 0.006; 12 weeks, *p* = 0.006). This shows that for 6 weeks of treatment, Restake treatment is able to significantly reduce anxiety, depression, and stress as compared to the Placebo group (Figure 2). It was noted that there was a significant decrease in serum cortisol levels in the Restake group compared to the placebo group after only 6 weeks. Relative to the baseline values, serum cortisol levels in the Restake group significantly decreased at both 6 weeks and 12 weeks, with reductions of −4.4% and −5.5%, respectively, compared to smaller reductions of 0.7% and 0.8% in the placebo group (6 weeks, *p* < 0.001; 12 weeks, *p* < 0.001) (Figure 2). ACTH levels also showed significant reductions in the Restake group, with decreases of −10.5% at 6 weeks and −8.1% at 12 weeks, compared to minimal changes of 0.3% and −0.2% in the placebo group (6 weeks, *p* < 0.001; 12 weeks, *p* < 0.001). This demonstrates that Restake effectively alleviates stress biomarkers within just 6 weeks.

**TABLE 2:** Stress and anxiety assessment by serum cortisol, ACTH level, STAI‐S, PSS, HAM‐A, and BDI and changes (%) after 12‐week intervention from baseline.

		STAIS Score	PSS Score	HAM‐A Score	BDI Score	Cortisol (ng/mL)	ACTH (pg/mL)
		Mean ± SD	Mean ± SD	Mean ± SD	Mean ± SD	Mean ± SD	Mean ± SD
Placebo	Baseline	49.2 ± 2.0	20.6 ± 1.8	8.9 ± 1.6	8.6 ± 1.7	168.8 ± 12.0	49.1 ± 8.1
	Week 6	47.7 ± 1.9	19.3 ± 1.5	8.2 ± 1.5	8.6 ± 1.4	169.0 ± 11.5	49.0 ± 7.5
	Week 12	46.7 ± 2.7	18.5 ± 1.5	7.7 ± 1.5	8.0 ± 1.5	168.9 ± 9.8	48.7 ± 7.3
^a^ *p*‐value		<0.001	<0.001	<0.001	0.020	0.980	0.330
Changes from baseline to Week 12 (%)		−4.9 ± 1.2	−10.1 ± 1.9	−13.1 ± 2.6	−4.1 ± 2.6	0.8 ± 0.9	−0.2 ± 0.9
Restake	Baseline	48.8 ± 2.0	21.2 ± 1.5	9.1 ± 1.9	9.2 ± 2.3	169.0 ± 16.1	48.3 ± 8.1
	Week 6	46.2 ± 2.4	18.8 ± 1.4	7.6 ± 1.7	7.7 ± 2.3	162.9 ± 11.7	44.5 ± 8.5
	Week 12	45.2 ± 2.1	18.5 ± 2.4	7.1 ± 1.6	7.7 ± 2.1	162.6 ± 11.0	45.3 ± 8.3
^a^ *p*‐value		<0.001	<0.001	<0.001	<0.001	0.007	<0.001
Changes from baseline to Week 12 (%)		−8.7 ± 0.8	−17.8 ± 1.8	−33.5 ± 5.5	−16.8 ± 3.7	−5.5 ± 1.2	−8.1 ± 1.4
^b^ *p*‐value		0.011	0.006	0.002	0.008	<0.001	<0.001

*Note*: ^a^ *p*‐value analysed by one way repeated measured ANOVA within group analysis (baseline, Week 6, and Week 12).

^b^ *p*‐value analysed by independent sample T‐test for between group analysis (Placebo and Restake).

### 3.3 Secondary outcome

**FIGURE 3:**
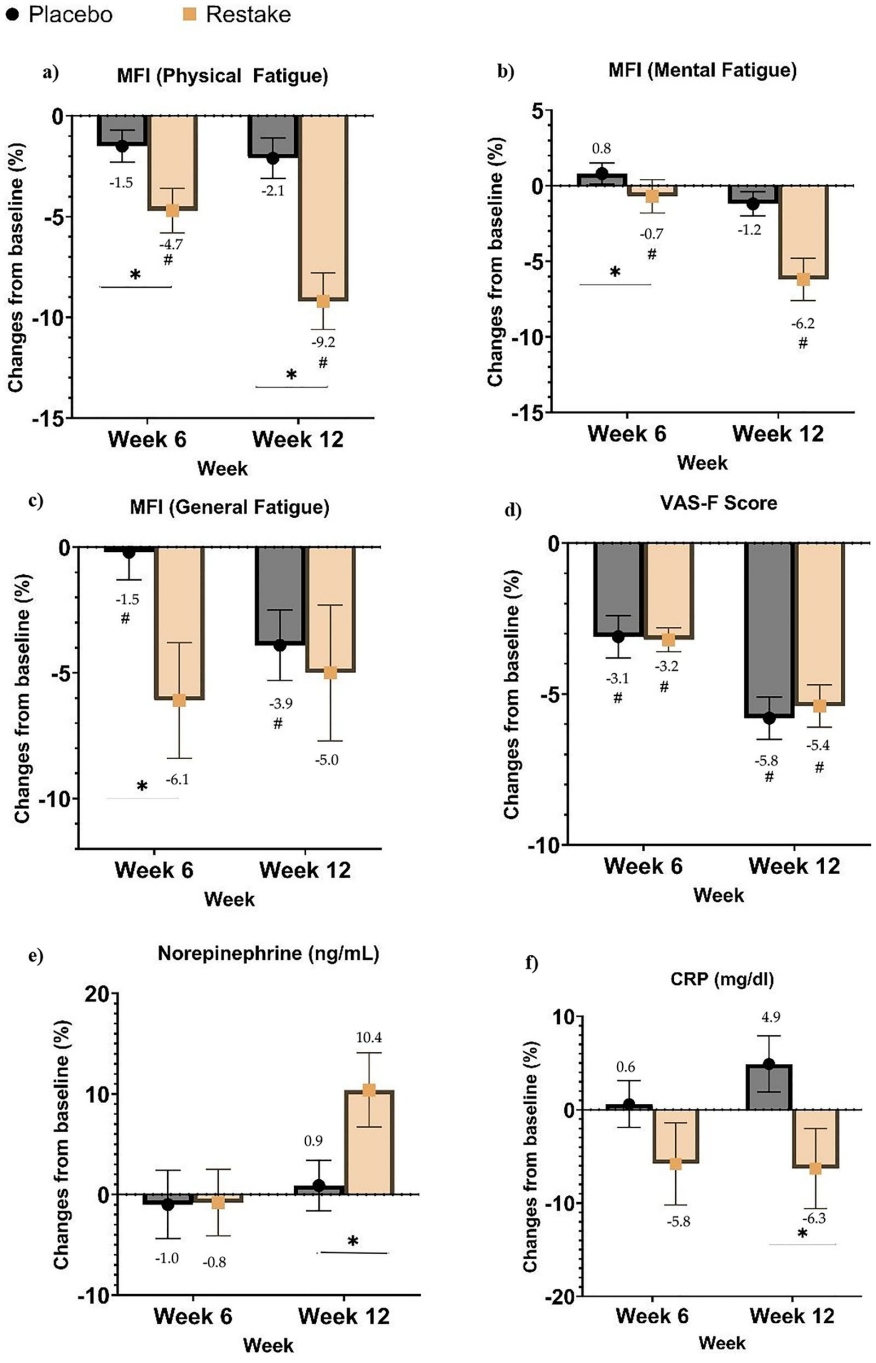
Percentage changes from baseline in MFI (a) physical (b) general, (c) mental, (d) VAS‐F, (e) NE, and (f) CRP level. * Indicates significant difference between the Placebo and the Restake groups, while # indicates significant difference within the group.

Within group analysis for fatigue assessments (VASF and MFI) recorded a significant difference over time points for both the Restake and the Placebo group (*p* < 0.05), as shown in Table 3. Restake showed the greatest reduction in physical fatigue, followed by mental and general fatigue, as shown in Figure 3. At 6 weeks, physical fatigue dropped by −4.7% in the Restake group versus −1.5% in the placebo (*p* = 0.027), with even greater reductions at 12 weeks (−9.2% vs. −2.1%, *p* < 0.001). Mental fatigue also improved, with a −1.2% reduction at 6 weeks compared to a 0.8% increase in the placebo group (*p* = 0.043). By 12 weeks, the Restake group saw a −6.2% reduction, though the difference was not statistically significant (*p* = 0.768). General fatigue decreased significantly in the Restake group at 6 weeks (−3.9% vs. −0.2%, *p* = 0.043). At 12 weeks, both groups showed reductions (−5.0% for Restake and −6.1% for placebo), though the significant effect between groups was not maintained (p = 0.768). Fatigue measured by VAS‐F showed reductions in both groups at 6 and 12 weeks, but differences were not statistically significant. These results highlight Restake’s strong impact on physical fatigue, with additional benefits for mental and general fatigue. NE levels at 12 weeks showed a significant increase in the Restake group, with a change of +10.4%, while the placebo group exhibited a decrease of −0.8% (*p* = 0.033). Relative to the value at baseline, CRP levels at 12 weeks demonstrated a significant decrease in the Restake group, with a reduction of −6.3%, whereas the placebo group showed an increase of +4.9% (*p* = 0.042) (Figure 3). The results imply that 500 mg/day treatment of Restake is effective in reducing serum NE and CRP as fatigue biomarkers in 12 weeks (Figure 3).

**TABLE 3:** Fatigue assessment by CRP, NE Level, VAS‐F, and MFI and changes (%) after 12‐week intervention from baseline.

		VASF score	MFI score (physical fatigue)	MFI score (mental fatigue)	MFI score (general fatigue)	Norepinephrine (ng/mL)	CRP (mg/dL)
		Mean ± SD	Mean ± SD	Mean ± SD	Mean ± SD	Mean ± SD	Mean ± SD
Placebo	Baseline	112.5 ± 4.2	13.4 ± 1.1	13.3 ± 1.4	14.3 ± 1.4	0.75 ± 0.11	0.06 ± 0.01
	Week 6	109.2 ± 4.2	13.1 ± 1.1	13.3 ± 1.3	14.1 ± 1.4	0.73 ± 0.15	0.06 ± 0.01
	Week 12	106.4 ± 4.1	13.0 ± 1.4	13.0 ±1.3	13.4 ± 0.9	0.73 ± 0.09	0.07 ± 0.01
^a^ *p*‐value		**<0.001**	0.056	0.217	**0.006**	0.713	0.290
Changes from baseline to Week 12 (%)		−5.8 ± 0.7	−2.1 ±1.0	−0.7 ± 1.1	−6.1 ± 2.3	−0.8 ± 3.3	4.9 ± 3.0
Restake	Baseline	113.4 ± 3.9	13.3 ± 1.4	13.3 ± 1.6	14.0 ± 1.5	0.71 ± 0.07	0.07 ± 0.01
	Week 6	110.0 ± 3.2	12.7 ± 1.1	13.3 ± 1.5	13.3 ± 1.4	0.74 ± 0.10	0.06 ± 0.01
	Week 12	107.7 ± 3.0	12.2 ± 1.1	12.7 ± 1.5	13.8 ± 1.8	0.82 ± 0.25	0.06 ± 0.01
^a^ *p*‐value		**<0.001**	**0.016**	**<0.001**	0.140	0.085	0.173
Changes from baseline to Week 12 (%)		−5.4 ± 0.7	−9.2 ± 1.4	−6.2 ± 1.4	−5.0 ±2.7	10.4 ± 3.7	−6.3 ± 4.3
^b^ *p*‐value		0.694	**<0.001**	0.768	0.768	**0.033**	**0.042**

*Note*: ^a^ *p*‐value analysed by one way repeated measured ANOVA within group analysis (baseline, Week 6, and Week 12).

^b^ *p*‐value analysed by independent sample T‐test for between group analysis (Placebo and Restake).

**FIGURE 4:**
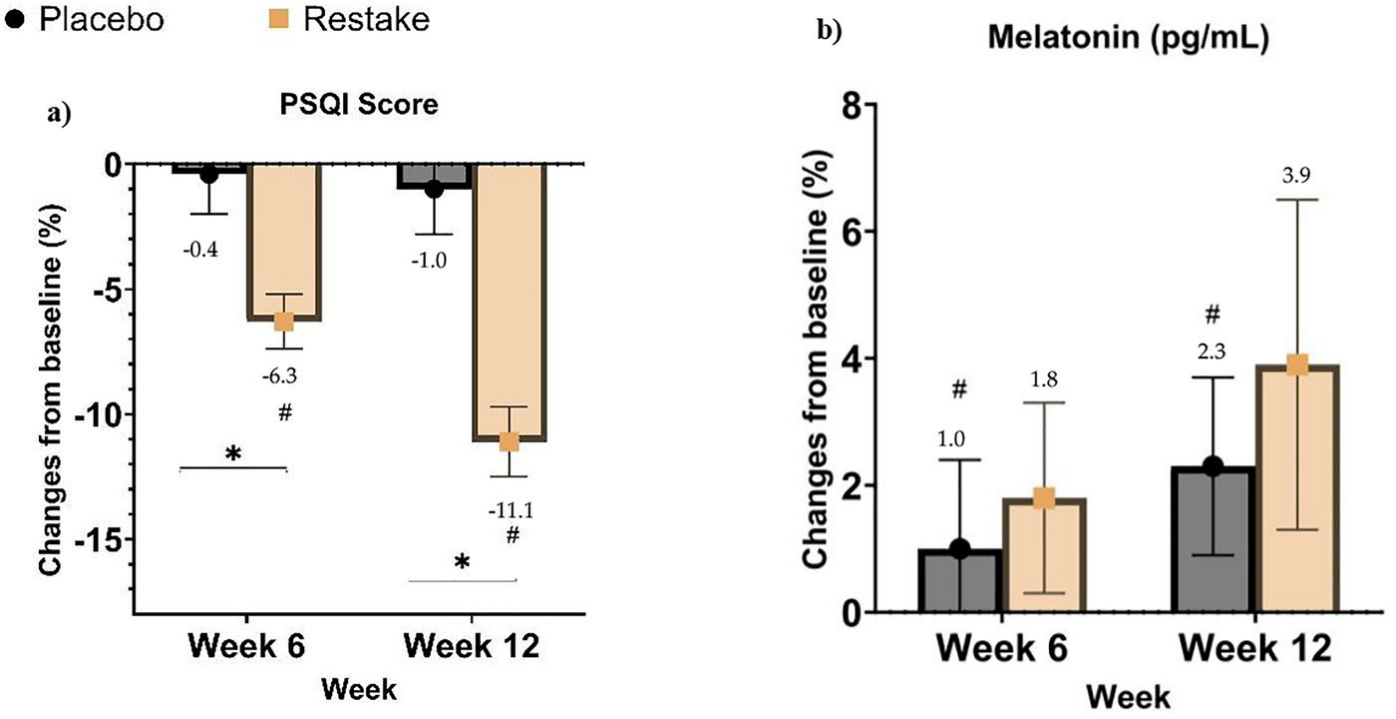
Percentage changes from baseline in (a) PSQI score and (b) melatonin level. * Indicates significant difference between the placebo and the Restake groups, while # indicates significant difference within the group.

Within group analysis, revealed a significant difference for the PSQI score for the Restake group and melatonin for the Placebo group (Table 4) over time (*p* < 0.05). As shown in Figure 4, the placebo group exhibited a 0.4% change of PSQI score, while the Restake group showed a −6.4% change (*p* = 0.005), indicating there is significant improvement in terms of overall sleep quality of the participants in the Restake group at Week 6. At 12 weeks, the placebo group showed a ‐1.0% change, and the Restake group showed a −11.1% change (*p* < 0.001). The effect of Restake on improving sleep quality was analyzed by melatonin level. Compared to the placebo group, Restake supplementation exhibited an increased morning melatonin trend after 12 weeks of intervention; however, there is no significant difference between the groups (Figure 4).

**TABLE 4:** Melatonin level and PSQI score and changes (%) after 12‐week intervention from baseline.

		PSQI score	Melatonin (pg/mL)
		Mean ± SD	Mean ± SD
Placebo	Baseline	21.9 ± 2.8	13.0 ± 0.6
	Week 6	21.6 ± 2.3	12.9 ± 0.6
	Week 12	21.4 ± 2.1	13.4 ± 0.6
^a^ *p*‐ value		0.332	**0.034**
Changes from baseline to Week 12 (%)		−1.0 ± 1.8	2.3 ± 1.4
Restake	Baseline	21.5 ± 2.7	12.2 ± 0.6
	Week 6	20.3 ± 2.1	12.6 ± 0.6
	Week 12	19.6 ± 2.1	12.7 ± 0.6
^a^ *p*‐ value		**<0.001**	0.112
Changes from baseline to Week 12 (%)		−11.1 ± 1.4	3.9 ± 2.6
^b^ *p*‐value		**<0.001**	0.597

*Note*: ^a^ *p*‐value analysed by one way repeated measured ANOVA within group analysis (baseline, Week 6, and Week 12).

^b^ *p*‐value analysed by independent sample T‐test for between group analysis (Placebo and Restake).

### 3.4 Safety and Tolerability

All measured safety parameters, including liver function, renal function, hematological indices, electrolyte levels, and blood glucose level, remained within the normal reference ranges throughout the study period. While some parameters exhibited statistically significant changes over time, these fluctuations were minor and not clinically relevant (Supplementary Data 3, Table 1).

## 4 Discussion

Stress and anxiety have been identified as promoters of multiple disease conditions, including neurodegenerative conditions such as Alzheimer's disease, cardiovascular related health issues, and non‐communicable disease such as diabetes and obesity (Salve et al. 2019). Numerous studies demonstrated the adaptogenic effect of medicinal mushrooms, which could be attributed to their antioxidant, anti‐inflammatory, cholinesterase, and potent neuroprotective activities (Liuzzi et al. 2023; Abitbol et al. 2022). This study is the first to evaluate the combination of five medicinal mushrooms as a potent and safe solution for stress, fatigue, and sleep improvement.

In the present study, participants in the treatment group demonstrated a statistically significant reduction in stress levels. Within group analysis for the primary outcome revealed a significant difference for both groups at different time points, which is baseline, Week 6, and Week 12. Restake significantly reduced anxiety (STAI‐S, HAM‐A) by Week 6 and improved BDI scores, suggesting a mood‐enhancing effect. Despite no significant differences in perceived stress levels between groups through the PSS test, the reductions in anxiety and depressive symptoms indicate substantial psychological benefits from Restake supplementation. These findings suggest that Restake supplementation may effectively modulate stress responses, alleviate anxiety symptoms, and reduce depressive manifestations, supporting its potential role in enhancing psychological resilience and emotional well‐being within just 6 weeks.

ACTH and serum cortisol level are the most used biomarkers to assess psychological stress associated with the HPA axis system (Noushad et al. 2021). When the human body perceives stress, ACTH will trigger the production of cortisol to prepare the body for a fight or flight response. A persistent elevated serum cortisol level throughout the day can be considered an alarming condition; a high level of cortisol could lead to HPA axis overactivation and circadian rhythm imbalance, consequently causing sleep disruption and chronic fatigue (Hinds and Sanchez 2022; Soler‐López et al. 2024). It is important to note that the antidepressant effect of Restake is likely multifactorial, as each medicinal mushroom in its blend has been shown to modulate the HPA axis while also influencing neurotransmitter activity and neuroplasticity (Noushad et al. 2021).

Physical fatigue showed significant improvements at both 6 weeks (*p* = 0.027) and 12 weeks (*p* < 0.001), while general and mental fatigue, as well as VAS‐F scores, showed early improvements but were not sustained over time, likely due to its β‐glucan content supporting energy metabolism and act as anti‐inflammatory activities (Muroya et al. 2025; Zhang et al. 2024), while its broader effects on fatigue may require prolonged use or higher doses. Besides the HPA system, there are other systems in our body that collectively regulate the level of stress, for example, autonomic nervous system (ANS) and the immune system. Higher CRP levels increased the release of pro‐inflammatory cytokines (IL‐6 and TNF‐α), which affect central nervous system (CNS) signaling, leading to fatigue, mood changes, and reduced motivation (Renner et al. 2022; Wang et al. 2021). It is noted that serum CRP levels showed a significant elevation at baseline (Table 1), indicating low‐grade inflammation. Treatment with Restake significantly reduced serum CRP level at 12 weeks (*p* = 0.042), suggesting anti‐inflammatory effects, supporting its role in mitigating inflammation‐associated, persistent fatigue. Therefore, the present study provides a clear mechanistic approach to reducing the stress‐induced fatigue through anti‐inflammatory activities and the HPA system. β‐glucan, the key active compound in Restake was reported to modulate immune responses by interacting with dectin‐1 receptors on immune cells, reducing NF‐kB signaling and inflammatory cytokine production (Brown and Gordon 2005). Additionally, *L. edodes* has been reported to reduce inflammation linked to chronic stress, potentially contributing to the 19.3% reduction in general fatigue. *G. frondosa* supports glucose metabolism and immune health, with β‐glucan stabilizing energy levels and reducing cortisol, which may explain the 7.9% ACTH reduction and physical fatigue improvements. *H. erinaceus* promotes neural health by stimulating nerve growth factor (NGF), potentially aiding cognitive function and contributing to the 33.5% anxiety reduction (HAM‐A).

Fatigue is commonly characterized by dysregulation of autonomic balance, manifesting as heightened sympathetic activity and attenuated parasympathetic activity, causing variations in circulating concentrations of epinephrine and norepinephrine (Son et al. 2016). NE levels showed a significant increase in the Restake group at 12 weeks compared to a decrease in the placebo group, indicating potential effects on the sympathetic nervous system or neuroendocrine pathways. The observed effect is potentially mediated by the bioactive compounds in the mushroom blend, such as hericenones and erinacines from *H. erinaceus*, which are known to influence neurogenesis and neurotransmitter activity (Saitsu et al. 2019).

It is clear that insomnia and depression are intimately related. The reduction trends across multiple PSQI components indicate a holistic improvement (11.1%) in overall sleep quality, with a significant reduction in daytime dysfunction. While other parameters did not reach statistical significance, the overall PSQI score trend suggests enhanced sleep efficiency and reduced sleep disturbances, which are crucial for long‐term sleep health. Melatonin, a hormone produced by the pineal gland in response to darkness, regulates the sleep‐wake cycle (Reiter et al. 2017). This study observed an increasing trend in melatonin levels in the treatment group, suggesting a potential modulatory effect. However, melatonin secretion varies among individuals due to factors like age, lifestyle, and circadian rhythms, which may affect statistical significance (Cipolla‐Neto et al. 2014). Since melatonin peaks at night, evening blood sampling would provide more accurate data than morning samples. Despite the lack of statistical significance, even small melatonin increases can enhance sleep quality, circadian regulation, and stress reduction (Reiter et al. 2017; Cipolla‐Neto et al. 2014; Zisapel 2018).

These findings highlight the adaptogenic effects of Restake in regulating the HPA axis, reducing inflammation, and supporting neuroendocrine balance. Medicinal mushrooms contain bioactive compounds like polysaccharides, triterpenes, and β‐glucan, which modulate the stress response and immune function (Wasser 2014; Patel et al. 2021). The results align with evidence supporting adaptogens in promoting neuroendocrine balance and reducing stress‐induced hormonal dysregulation (Tsigos et al. 2002). The antidepressant‐like effects of medicinal mushrooms further support their role in improving mental health outcomes (Wasser 2014).

## 5 Conclusions

The findings of this study demonstrate that Restake supplementation significantly improves sleep quality, reduces physical and general fatigue, and alleviates anxiety and depressive symptoms, with these effects being more pronounced compared to the placebo. The significant reductions in cortisol and ACTH levels further support the adaptogenic potential of the mushroom blend in modulating the hypothalamic‐pituitary‐adrenal (HPA) axis, a key regulator of the body's stress response. The improvement in the inflammation markers, including CRP and NE level, and the increase in melatonin level supported by the significant improvement in PSQI score indicated the effectiveness of Restake supplementation in managing moderate to severe fatigue and sleep, respectively. These results suggest that the mushroom blend offers a natural and effective intervention for managing stress‐related health issues, particularly those involving dysregulated stress hormones. Future studies should explore its long‐term effects and applicability in clinical populations with chronic stress or related disorders.

## Author Contributions

Conceptualization: Teik Kee Leo and Mohamad Shazeli Che Zain. Methodology: Ahmad Safiyyu'd‐din Hisamuddin and Faiqah Ramli. Software: Tze Yan Lee. Validation: Le Jie Lee. Formal analysis: Mei Szin Wong and Mohd Raili Suhaili. Investigation: Tze Yan Lee. Resources: Mei Szin Wong and Mohd Raili Suhaili, and Tze Yan Lee. Data curation: Ahmad Safiyyu'd‐din Hisamuddin. Writing – original draft preparation: Ahmad Safiyyu'd‐din Hisamuddin. Writing – review and editing: Faiqah Ramli. Supervision: Faiqah Ramli. Project administration: Le Jie Lee. Funding acquisition: Faiqah Ramli. All authors have read and agreed to the published version of the manuscript.

## Funding

This study was funded by the NexusWise Sdn Bhd.

## Ethics Statement

This study protocol was approved by the SEGi Research Ethics Committee (SEGiEC/StR/FOM/379/2024‐2025) and was conducted in accordance with the Declaration of Helsinki and Good Clinical Practice Guidelines.

## Supporting information




**Supporting Information**: brb371193‐supp‐0001‐SuppMatData1.pdf

Supporting Information: brb371193‐supp‐0002‐SuppMatData2.pdf

Supporting Information: brb371193‐supp‐0003‐SuppMatData3.pdf

## Data Availability

The data presented in this study are available upon request from the first author.
