# Supplementary material for: Adaptogenic Effects of Mushroom Blend Supplementation on Stress, Fatigue, and Sleep: A Randomised, Double‐Blind, and Placebo‐Controlled Trial
Source: Brain Behav. 2026 Jan 15;16(1):e71193. doi: 10.1002/brb3.71193 (PMC12808922; doi:10.1002/brb3.71193)
Supplement: Supplementary file 1 — Supporting Information: brb371193‐supp‐0001‐SuppMatData1.pdf [file BRB3-16-e71193-s002.pdf]

www.nexuswise.com

NEXUSWISE
Health Ingredients · Wise Solutions

Address
Unit C-G-08 (Lobby 4).
Block C Damansara Intan,
e-Business Park, Jalan SS 20/27,
47400 Petaling Jaya,Selangor, Malaysia.

Contact
Tel: +603 7728 1637 / 1590
Fax: +603 7728 5164

Email
info@nexuswise.com

To whom it may concern,

DECLARATION OF INGREDIENT LIST

We, “Nexus Wise Sdn Bhd” would like to confirm that we manufacture and market RESTAKE.

We certify that we have not added to it any substance which does not comply with these criteria, and that this product is derived from mushroom extract and mushroom powder.

Product description:

Appearance: Brown powder

Table 1: Ingredients listed in Restake

1.	Mushroom Blend Extract	22.5%
2.	Mushroom Blend Powder	10%
3.	Arabic Gum	33.75%
4.	Beta-Cyclodextrin	33.75%
	Total	100%

Kai Shin/ QC Exec,

Nexus Wise Sdn. Bhd.
