# Supplementary material for: Adaptogenic Effects of Mushroom Blend Supplementation on Stress, Fatigue, and Sleep: A Randomised, Double‐Blind, and Placebo‐Controlled Trial
Source: Brain Behav. 2026 Jan 15;16(1):e71193. doi: 10.1002/brb3.71193 (PMC12808922; doi:10.1002/brb3.71193)
Supplement: Supplementary file 2 — Supporting Information: brb371193‐supp‐0002‐SuppMatData2.pdf [file BRB3-16-e71193-s003.pdf]

www.nexuswise.com

NEXUSWISE
Health Ingredients · Wise Solutions

Address
Unit C-G-08 (Lobby 4).
Block C Damansara Intan,
e-Business Park, Jalan SS 20/27,
47400 Petaling Jaya,Selangor, Malaysia.

Contact
Tel: +603 7728 1637 / 1590
Fax: +603 7728 5164

Email
info@nexuswise.com

METHOD OF ANALYSIS FOR RESTAKE

**Product Name**	**RESTAKE**
**Batch No**	**RTK-250810**
**Analysis**	**1) Yeast Beta Glucan**<br>**2) Total Polysaccharide**
**Method Analysis**	**1) In-house (Based on Mushroom Yeast Beta-glucan Assay Procedure) Code: K-YBGL**<br>**2) In-house (Phenol-sulphuric acid method)**
**Date of analysis**	**21st August 2025**

Table 1: Result of yeast beta glucan and total polysaccharide in Restake

Test	Result	Unit	Method Reference
Yeast Beta Glucan	32.02 ± 0.25	% w/w	In-house (Based on Mushroom and Yeast Beta-Glucan Assay Procedure)
Total Polysaccharide	34.53 ± 2.77	% w/w	In-house (Phenol-sulphuric acid method)

Prepared by

Ahmad Safiyyu'd-din

R&D Executive

Verify by

Ts. ChM. Dr. Faiqah Ramli

Assistant Manager Research and Development
