# Supplementary material for: Adaptogenic Effects of Mushroom Blend Supplementation on Stress, Fatigue, and Sleep: A Randomised, Double‐Blind, and Placebo‐Controlled Trial
Source: Brain Behav. 2026 Jan 15;16(1):e71193. doi: 10.1002/brb3.71193 (PMC12808922; doi:10.1002/brb3.71193)
Supplement: Supplementary file 3 — Supporting Information: brb371193‐supp‐0003‐SuppMatData3.pdf [file BRB3-16-e71193-s001.pdf]

Table 1: Safety Assessment of Liver, Renal, Diabetic, Electrolyte, and Hematological Parameters from Baseline, Week 6, and Week 12

Measurements	Visit	Placebo (n=25)	Restake® (n=25)	p-value
**Liver Function**				
Total Protein (g/L)	Baseline	77.6 ± 3.6	76.9 ± 3.1	0.553
	Week 6	76.3 ± 3.5	77.4 ± 3.2	0.640
	Week 12	78.4 ± 3.4	75.7 ± 3.3	0.097
Albumin (g/L)	Baseline	40.8 ± 2.8	39.6 ± 2.5	0.052
	Week 6	43.5 ± 2.9	41.3 ± 2.6	0.007
	Week 12	40.7 ± 2.7	42.4 ± 2.5	0.057
Globulin (g/L)	Baseline	37.7 ± 3.7	37.4 ± 2.9	0.964
	Week 6	33.7 ± 3.8	35.5 ± 3.0	0.035
	Week 12	36.8 ± 3.6	34.5 ± 2.8	0.088
A/G Ratio	Baseline	1.1 ± 0.2	1.1 ± 0.1	0.282
	Week 6	1.4 ± 0.2	1.2 ± 0.1	<0.001
	Week 12	1.1 ± 0.1	1.3 ± 0.1	0.002
Total Bilirubin (umol/L)	Baseline	9.0 ± 5.8	8.0 ± 2.1	0.412
	Week 6	7.7 ± 5.2	7.7 ± 2.0	0.987
	Week 12	8.5 ± 5.4	8.1 ± 2.1	0.745
AST (SGOT) (U/L)	Baseline	17.9 ± 3.8	20.4 ± 9.7	0.266
	Week 6	22.7 ± 4.2	22.0 ± 8.9	0.751
	Week 12	20.7 ± 3.9	19.1 ± 9.2	0.318
ALT (SGPT) (U/L)	Baseline	20.9 ± 12.6	24.4 ± 16.5	0.345
	Week 6	23.9 ± 12.2	27.8 ± 17.3	0.366

	Week 12	21.4 ± 12.5	21.0 ± 15.6	0.926
ALP (U/L)	Baseline	82.6 ± 21.1	85.4 ± 19.8	0.417
	Week 6	80.1 ± 20.3	84.7 ± 20.2	0.270
	Week 12	81.4 ± 19.5	82.3 ± 19.9	0.577
GGT (U/L)	Baseline	20.7 ± 6.4	21.9 ± 7.3	0.461
	Week 6	21.1 ± 5.8	20.6 ± 6.7	0.487
	Week 12	21.4 ± 6.2	21.0 ± 6.9	0.660
**Renal Function**				
Urea (mmol/L)	Baseline	3.7 ± 0.7	3.4 ± 0.7	0.059
	Week 6	3.7 ± 0.9	3.6 ± 1.0	0.353
	Week 12	3.7 ± 0.7	4.9 ± 5.9	0.665
Creatinine (umol/L)	Baseline	70.6 ± 14.1	65.6 ± 9.6	0.101
	Week 6	66.8 ± 13.6	64.7 ± 9.1	0.445
	Week 12	69.4 ± 14.4	65.8 ± 12.8	0.299
Estimated GFR (mL/min/1.73m²)	Baseline	87.3 ± 12.2	90.5 ± 17.9	0.397
	Week 6	87.2 ± 14.7	90.0 ± 14.0	0.417
	Week 12	82.4 ± 10.8	86.6 ± 17.4	0.386
Uric Acid (umol/L)	Baseline	345.2 ± 85.4	332.1 ± 78.3	0.407
	Week 6	0.36 ± 0.09	0.32 ± 0.08	0.063
	Week 12	0.31 ± 0.09	0.36 ± 0.08	0.096
Calcium (mmol/L)	Baseline	2.34 ± 0.09	2.35 ± 0.08	0.298
	Week 6	2.28 ± 0.09	2.25 ± 0.09	0.126
	Week 12	2.32 ± 0.10	2.27 ± 0.08	0.079
Adjusted Calcium (mmol/L)	Baseline	2.32 ± 0.07	2.31 ± 0.06	0.215

	Week 6	2.18 ± 0.08	2.25 ± 0.11	0.011
	Week 12	2.33 ± 0.09	2.23 ± 0.08	0.001
Inorganic Phos (mmol/L)	Baseline	1.17 ± 0.21	1.19 ± 0.18	0.375
	Week 6	1.13 ± 0.15	1.18 ± 0.13	0.178
	Week 12	1.13 ± 0.14	1.12 ± 0.16	0.578
**Diabetic Studies**				
Fasting Glucose (mmol/L)	Baseline	5.2 ± 0.6	5.1 ± 0.5	0.308
	Week 6	4.9 ± 0.4	5.1 ± 1.6	0.480
	Week 12	4.8 ± 0.4	5.3 ± 1.4	0.104
**Electrolytes**				
Sodium (mmol/L)	Baseline	139.7 ± 1.8	139.3 ± 1.9	0.675
	Week 6	137.7 ± 2.1	139.0 ± 2.9	0.450
	Week 12	139.3 ± 2.9	138.1 ± 3.7	0.661
Chloride (mmol/L)	Baseline	102.8 ± 1.8	102.4 ± 1.9	0.663
	Week 6	102.0 ± 1.7	101.1 ± 2.4	0.065
	Week 12	103.0 ±1.8	102.1 ± 2.1	0.100
Potassium (mmol/L)	Baseline	4.5 ± 0.3	4.5 ± 0.3	0.331
	Week 6	4.6 ± 0.4	4.5 ± 0.4	0.367
	Week 12	4.4 ± 0.2	4.5 ± 0.3	0.087
**Hematology**				
Hemoglobin (g/L)	Baseline	133.6 ± 15.7	131.3 ± 10.2	0.452
	Week 6	130.9 ± 15.2	132.3 ± 9.7	0.750
	Week 12	135.4 ± 17.9	139.0 ± 16.5	0.286
RBC ($x10^{12}$/L)	Baseline	4.8 ± 0.5	4.8 ± 0.4	0.836
	Week 6	4.8 ± 0.4	4.8 ± 0.4	0.793

	Week 12	4.9 ± 0.6	5.1 ± 0.5	0.151
RDW (%)	Baseline	13.4 ± 1.1	13.2 ± 1.0	0.194
	Week 6	14.0 ± 1.8	13.8 ± 1.4	0.515
	Week 12	13.6 ± 1.7	14.2 ± 1.5	0.286
PCV (L/L)	Baseline	41.1 ± 3.9	40.7 ± 3.5	0.429
	Week 6	0.40 ± 0.03	0.40 ± 0.02	0.963
	Week 12	0.41 ± 0.05	0.42 ± 0.05	0.248
MCH (pg)	Baseline	28.1 ± 2.4	27.9 ± 2.3	0.282
	Week 6	27.2 ± 3.0	27.5 ± 2.1	0.519
	Week 12	27.6 ± 2.4	27.3 ± 2.7	0.785
MCV (fL)	Baseline	85.6 ± 4.8	84.9 ± 4.5	0.250
	Week 6	83.1 ± 6.3	83.0 ± 5.2	0.981
	Week 12	83.7 ± 5.4	82.9 ± 6.4	0.696
MCHC (g/L)	Baseline	328.2 ± 12.5	326.7 ± 11.8	0.940
	Week 6	326.4 ± 14.2	328.9 ± 11.5	0.322
	Week 12	328.5 ± 10.1	328.3 ± 11.6	0.811
Platelet Count (x10^9/L)	Baseline	311.2 ± 69.6	315.7 ± 87.8	0.840
	Week 6	312.6 ± 86.9	313.1 ± 79.5	0.825
	Week 12	322.6 ± 67.9	308.4 ± 110.1	0.576
Total WCC (x10^9/L)	Baseline	7.3 ± 1.5	7.4 ± 1.9	0.911
	Week 6	7.3 ± 1.6	7.5 ± 1.8	0.561
	Week 12	7.2 ± 1.3	7.7 ± 1.7	0.184
Neutrophils (x10^9/L)	Baseline	4.04 ± 1.08	4.07 ± 1.27	0.975
	Week 6	3.93 ± 0.93	4.22 ± 1.30	0.411
	Week 12	4.02 ± 0.97	4.62 ± 1.41	0.062

Lymphocytes ($x10^9$/L)	Baseline	2.47 ± 0.63	2.49 ± 0.82	0.612
	Week 6	2.41 ± 0.60	2.53 ± 0.87	0.427
	Week 12	2.42 ± 0.63	2.38 ± 0.54	0.962
Monocytes ($x10^9$/L)	Baseline	0.48 ± 0.13	0.51 ± 0.17	0.630
	Week 6	0.53 ± 0.21	0.54 ± 0.20	0.637
	Week 12	0.47 ± 0.13	0.46 ± 0.13	0.874
Eosinophils ($x10^9$/L)	Baseline	0.26 ± 0.29	0.20 ± 0.09	0.311
	Week 6	0.35 ± 0.41	0.19 ± 0.09	0.064
	Week 12	0.18 ± 0.09	0.19 ± 0.10	0.552
Basophils ($x10^9$/L)	Baseline	0.05 ± 0.02	0.05 ± 0.02	0.764
	Week 6	0.05 ± 0.02	0.05 ± 0.02	0.307
	Week 12	0.04 ± 0.02	0.05 ± 0.02	0.046
ESR (mm/hr)	Baseline	18.3 ± 11.8	28.9 ± 16.8	0.008
	Week 6	23.7 ± 14.2	17.3 ± 9.9	0.131
	Week 12	20.7 ± 14.0	16.1 ± 9.6	0.218
